# Non-Surgical Breast-Conserving Treatment (KORTUC-BCT) Using a New Radiosensitization Method (KORTUC II) for Patients with Stage I or II Breast Cancer

**DOI:** 10.3390/cancers7040891

**Published:** 2015-11-17

**Authors:** Yasuhiro Ogawa, Kei Kubota, Nobutaka Aoyama, Tomoaki Yamanishi, Shinji Kariya, Norihiko Hamada, Munenobu Nogami, Akihito Nishioka, Masahide Onogawa, Mitsuhiko Miyamura

**Affiliations:** 1 Hyogo Prefectural Kakogawa Medical Center, Kakogawa, Hyogo 675-8555, Japan; 2 Department of Diagnostic Radiology & Radiation Oncology, Medical School, Kochi University, Nankoku, Kochi 783-8505, Japan; kubotak@kochi-u.ac.jp (K.K.); jm-aoyama_nobutaka@kochi-u.ac.jp (N.A.); yamanist@kochi-u.ac.jp (T.Y.); kariyas@kochi-u.ac.jp (S.K.); hamadan@kochi-u.ac.jp (N.H.); aznogami@mopera.net (M.N.); nishiokaa@kochi-u.ac.jp (A.N.); 3 Department of Pharmacy, Medical School Hospital, Kochi University, Nankoku, Kochi 783-8505, Japan; jm-ma_ono@kochi-u.ac.jp (M.O.); miyamus@kochi-u.ac.jp (M.M.)

**Keywords:** hydrogen peroxide, radiosensitizer, sodium hyaluronate, radiotherapy, KORTUC, radiation therapy

## Abstract

The purpose of the present study was to establish a non-surgical breast-conserving treatment (BCT) using KORTUC II radiosensitization treatment. A new radiosensitizing agent containing 0.5% hydrogen peroxide and 0.83% sodium hyaluronate (a CD44 ligand) has been developed for intra-tumoral injection into various tumors. This new method, named KORTUC II, was approved by our local ethics committee for the treatment of breast cancer and metastatic lymph nodes. A total of 72 early-stage breast cancer patients (stage 0, 1 patient; stage I, 23; stage II, 48) were enrolled in the KORTUC II trial after providing fully informed consent. The mean age of the patients was 59.7 years. A maximum of 6 mL (usually 3 mL for tumors of less than approximately 3 cm in diameter) of the agent was injected into breast tumor tissue twice a week under ultrasonographic guidance. For radiotherapy, hypofraction radiotherapy was administered using a tangential fields approach including an ipsilateral axillary region and field-in-field method; the energy level was 4 MV, and the total radiation dose was 44 Gy administered as 2.75 Gy/fraction. An electron boost of 3 Gy was added three times. Treatment was well tolerated with minimal adverse effects in all 72 patients. No patients showed any significant complications other than mild dermatitis. A total of 24 patients under 75 years old with stage II breast cancer underwent induction chemotherapy (EC and/or taxane) prior to KORTUC II treatment, and 58 patients with estrogen receptor-positive tumors also received hormonal therapy following KORTUC II. The mean duration of follow-up as of the end of September 2014 was 51.1 months, at which time 68 patients were alive without any distant metastases. Only one patient had local recurrence and died of cardiac failure at 6.5 years. Another one patient had bone metastases. For two of the 72 patients, follow-up ended after several months following KORTUC II treatment. In conclusion, non-surgical BCT can be performed using KORTUC II, which has three major characteristics: imaging guidance; enzyme-targeting; and targeting of breast cancer stem cells via the CD44 receptor.

## 1. Introduction

We recently developed a new enzyme-targeting radiosensitization treatment using hydrogen peroxide for intratumoral injection (KORTUC II) and confirmed the safety and effectiveness of the treatment mainly for patients with locally advanced neoplasms [1,2]. Therefore, in this study, KORTUC II was used for non-surgical treatment of patients with stages I and II breast cancer. The purpose of the present study was to establish a non-surgical breast-conserving treatment (BCT) using KORTUC II radiosensitization treatment.

Recently, BCT has come to be considered a standard treatment method for patients with early-stage breast cancer [3]. BCT is composed of partial glandectomy (breast-conserving surgery) and postoperative radiation therapy. Approximately 25% of patients treated with breast-conserving surgery alone show local recurrence by five years after surgery. By adding postoperative radiation therapy, the local recurrence rates have been reported to decrease to approximately one-third. Radical mastectomy (Halsted operation), which had been performed as a standard treatment method until approximately 30 years ago, has almost disappeared today. It has become common consensus that the extent of surgery and surgical resection does not correlate with the survival rate of patients with breast cancer [4].

Though the extent of the surgical resection for breast cancer has rapidly decreased, axillary lymph node resection has been routinely performed. On the basis of evidence from Western countries that axillary lymph node dissection does not affect survival rates of patients with early-stage breast cancer [5], and that the rate of patients suffering from edema and/or functional dysfunction of the ipsilateral upper extremity as complications of axillary lymph node dissection is not relatively low, sentinel lymph node biopsy has become widespread in the last several years to avoid axillary lymph node dissection.

According to the strategy mentioned above, if all of the surgical procedures, including partial mastectomy and sentinel lymph node biopsy, could be omitted in standard therapy for breast cancer, both physical and mental invasiveness for patients should be greatly decreased, preserving cosmesis of the breast, and, moreover, medical costs will be less.

When we treat breast cancer without any surgical procedure, for local therapy, we need to depend on chemotherapy or radiotherapy or both. The overall chemosensitivity of breast cancer is relatively not high [6], and a primary chemo-radiotherapy trial led by the Japanese Clinical Oncology Group (JCOG) did not yield significant results in terms of local control of breast cancer and survival benefit for the patients [7]. In the chemoradiotherapy trial led by JCOG, it was actually a disappointment that KORTUC radiosensitization treatment was not used.

Radiosensitivity of the invasive component of breast cancer is reported to be relatively high. On the other hand, that of the non-invasive (intraductal) component is thought to be relatively low. The reason for this difference is that undifferentiated cells and cells showing active cell division are radiosensitive in mammalian cells, which has been shown by the Bergonie-Tribondeau^,^s law. Another major cause is that the well-differentiated intraductal component of breast cancer contains many antioxidative enzymes (peroxidase), and the radical reaction eliminates two-thirds of the effect of X-rays [8].

Accordingly, to avoid all surgical procedures in breast cancer therapy and to treat the primary lesion of breast cancer by radiotherapy alone as local treatment, the use of a heavy-particle beam such as a carbon beam whose effect is not decreased by hypoxia and/or radical reaction or the use of our new enzyme-targeting radiosensitization treatment KORTUC, which can increase partial oxygen pressure in hypoxic tumor tissue, and, at the same time, inactivate the antioxidative enzyme, peroxidase, is considered to be of utmost importance.

In this paper, the treatment results of non-surgical chemoradiosensitization (KORTUC II) therapy for 72 patients with early-stage breast cancer are reported. The treatment method is called KORTUC-BCT, and it is a new method of BCT applying KORTUC II without any surgical procedure.

## 2. Results

Treatment was well tolerated with minimal adverse effects in all 72 patients. No patients showed any significant complications (excluding mild dermatitis: Grade I, 45 patients; Grade II, 27 patients). Cosmetically, 62 (86.1%) of the 72 patients were evaluated as having excellent or good appearance of their ipsilateral breast. No Grade 3/4 acute toxicities were observed. Grade 3 telangiectasia was observed in 1.4% (1/72) of patients. A summary of the patients’ data is shown in Table 1.

**Table 1:** Summary of the patients’ data (asterisks * indicate the patients described in our previous paper [9]).

Case No.	Initial	Age	Site	cTNM	Induction Chemotherapy	Therapeutic Effect	Prognosis (y: years)
1 *	F.T.	88	Rt.E	cT2N1M0	-	cCR	NED > 6.0 y
2 *	Y.K.	78	Rt.D	cT2N0M0	-	cCR	NED > 7.0 y
3 *	T.N.	79	Lt.B & Rt.C	cT2N0M0 cT1cN0M0	-	cCR cCR	Dead at 6.5 y
4 *	Y.Y.	59	Lt.E	cT2N0M0	-	cCR	NED for 6.3 y
5	T.Y.	49	Rt.CD	cT2N0M0	EC × 4	cCR	NED for 7.1 y
6 *	H.M.	73	Lt.C	cT1cN0M0	-	cCR	NED for 6.3 y
7 *	O.W.	79	Lt.CD	cT2N0M0	-	cCR	NED for 3.5 y
8	K.K.	60	Rt.A	cT2N1M0	EC × 6	cCR	NED for 6.3 y
9 *	Y.F.	77	Rt.ADB	cT2N0M0	-	cCR	NED for 6.0 y
10 *	U.Y.	82	Rt.ABE	cT2N0M0	-	cCR	NED for 6.0 y
11 *	K.N.	63	Lt.C	cT2N0M0	-	cCR	NED for 6.2 y
12	T.K.	52	Rt.AB	cT2N1M0	EC × 4	cCR	NED for 5.9 y
13	H.T.	87	Lt.C	cT1cN0M0	-	cCR	NED for 5.8 y
14	N.S.	63	Rt.A	cT1cN0M0	-	cCR	NED for 5.8 y
15	K.M.	42	Rt.ECD	cTisN0M0	-	cCR	NED for 5.5 y
16	Y.J.	61	Rt.B	cT1cN0M0	-	cCR	NED for 5.4 y
17	S.C.	60	Rt.D	cT1cN0M0	-	cCR	NED for 5.3 y
18	C.N.	80	Rt.C	cT2N1M0	-	cCR	NED for 5.3 y
19	K.M.	43	Lt.B&E	cT2N0M0	FEC × 4	cCR	NED for 5.3 y
20	M.N.	42	Lt.C	cT2N0M0	FEC × 4	cCR	NED for 5.1 y
21 *	H.K.	77	Lt.AE	cT1cN1M0	-	cCR	NED for 5.1 y
22	Y.T.	47	Rt.AC	cT2N0M0	EC × 4	cCR	NED for 5.1 y
23	M.A.	47	Lt.BDE	cT2N0M0	EC × 4, TXT × 4	cCR	NED for 5.1 y
24	K.Y.	50	Rt.AB	cT1cN0M0	-	cCR	NED for 4.7 y
25	N.K.	67	Lt.D	cT2N0M0	EC × 4	cCR	NED for 5.0 y
26	S.S.	49	Lt.CD	cT2N0M0	EC × 4, TXT × 4	cCR	NED for 5.0 y
27	O.K.	43	Rt.C	cT1cN0M0	-	cCR	NED for 4.7 y
28	N.T.	76	Lt.C	cT1bN0M0	-	cCR	NED for 4.7 y
29	S.M.	45	Rt.E	cT2N0M0	EC × 4	cCR	NED for 4.5 y
30	O.A.	81	Rt.A	cT2N0M0	-	cCR	NED for 3.8 y
31	H.S.	83	Lt.B	cT2N0M0	-	cCR	NED for 4.1 y
32	T.S.	68	Rt.B	cT2N0M0	EC × 4	cCR	NED for 4.1 y
33	S.M.	29	Lt.C	cT1cN0M0	-	N.A.	N.A.
34	O.K.	63	Rt.CD	cT1cN0M0	-	cCR	NED for 3.6 y
35	I.N.	24	Lt.A	cT1cN0M0	-	cCR	NED for 3.5 y
36	N.K.	40	Rt.A	cT2N0M0	TC × 4	cCR	NED for 3.7 y
37	M.A.	59	Rt.E	cT1cN0M0	-	cCR	NED for 3.5 y
38	M.C.	68	Rt.A	cT2N0M0	EC × 4	cCR	NED for 3.6 y
39	Y.T.	37	Rt.D	cT1cN0M0	-	cCR	NED for 3.4 y
40	T.T.	49	Lt.A	cT2N0M0	-	cCR	NED for 3.1 y
41	W.C.	63	Lt.C	cT2N0M0	EC × 4	cCR	NED for 3.3 y
42	H.S.	70	Rt.A	cT2N1M0	EC × 4	cCR	NED for 3.1 y
43	K.E.	60	Lt.A	cT1cN1M0	EC × 4	cCR	NED for 2.9 y
44	Y.I.	44	Lt.C	cT2N0M0	-	cCR	NED for 2.7 y
45	H.I.	48	Rt.C	cT1cN0M0	-	cCR	NED for 2.5 y
46	F.K.	70	Rt.AB	cT1cN0M0	-	cCR	NED for 2.6 y
47	O.F.	55	Rt.D	cT2N1M0	EC × 4	cCR	NED for 2.8 y
48	K.M.	69	Lt.CD	cT2N0M0	-	cCR	NED for 4.1 y
49	T.K.	61	Rt.BD	cT2N1M0	EC × 4	cCR	Bone meta.
50	F.M.	61	Rt.C	cT2N1M0	EC × 4, TXT x 4	cCR	NED for 2.8 y
51	N.S.	59	Rt.C	cT1cN0M0	-	cCR	NED for 2.7 y
52	N.A.	78	Rt.A	cT1bN0M0	-	N.A.	N.A.
53	I.Y.	76	Rt.AB	cT2N0M0	-	cCR	NED for 2.2 y
54	Y.M.	63	Lt.A	cT2N0M0	-	cCR	NED for 2.2 y
55	Y.J.	50	Rt.A	cT2N1M0	FEC × 4,TXL × 4	cCR	NED for 2.0 y
56	S.M.	38	Lt.A	cT2N1M0	EC × 4, TXT × 4	cCR	NED for 2.6 y
57	Y.Y.	35	Lt.C	cT2N1M0	EC × 8	cCR	NED for 2.5 y
58	U.J.	56	Lt.CD	cT1cN0M0	-	cPR	Op. at 1 y
59	M.M.	79	Rt.ED	cT2N0M0	-	cCR	NED for 1.7 y
60	I.K.	39	Rt.C	cT1cN0M0	EC × 4	cCR	NED for 1.8 y
61	S.Y.	51	Rt.D	cT2N0M0	-	cCR	NED for 1.5 y
62	K.R.	46	Lt.C	cT1cN0M0	-	cCR	NED for 1.5 y
63	N.K.	47	Rt.BD	cT1cN1M0	EC × 4	cCR	NED for 1.4 y
64	H.S.	67	Rt.C	cT1cN0M0	-	cCR	NED for 1.4 y
65	O.Y.	74	Rt.C	cT2N0M0	-	cCR	NED for 1.4 y
66	Y.M.	63	Lt.BD	cT2N0M0	-	cCR	NED for 1.2 y
67	K.E.	58	Rt.AB	cT2N0M0	-	cCR	NED for 1.1 y
68	K.M.	54	Lt.A	cT1cN0M0	-	cCR	NED for 1.1 y
69	A.E.	43	Lt.C	cT2N0M0	-	cCR	NED for 1.0 y
70	S.M.	83	Rt.C	cT1cN0M0	-	cCR	NED for 1.2 y
71	S.T.	75	Lt.C	cT2N0M0	-	cCR	NED for 0.8 y
72	W.R.	39	Lt.AC	cT2N0M0	-	cCR	NED for 0.7 y

A total of 24 patients under 75 years old with stage II breast cancer undertook induction chemotherapy (EC and/or taxane) prior to KORTUC II treatment, and 58 patients with estrogen receptor-positive tumors were also started on hormone therapy following KORTUC II treatment. Of the 24 patients, four whose breast cancer failed to show significant reduction of SUV-max values on PET-CT images after four courses of EC chemotherapy also undertook four courses of tri-weekly taxane chemotherapy. After the treatment, they undertook KORTUC radio-sensitization therapy, and all of their clinical courses have been good so far.

On the PET-CT study and/or breast dynamic MRI performed after the KORTUC treatment, almost all patients showed clinically complete response (cCR) of their tumor, though the time period until achieving tumor disappearance varied for each individual patient, taking several months to approximately one year following KORTUC II treatment [9]. For two of the 72 patients, follow-up ended after several months following KORTUC II treatment.

Of the 71 lesions of 70 patients evaluated, 70 (98.6%) showed clinically complete response (cCR), and one (1.4%) showed a clinically partial response (cPR) and she undertook surgical resection at approximately one year following KORTUC II treatment.

**Figure 1:**
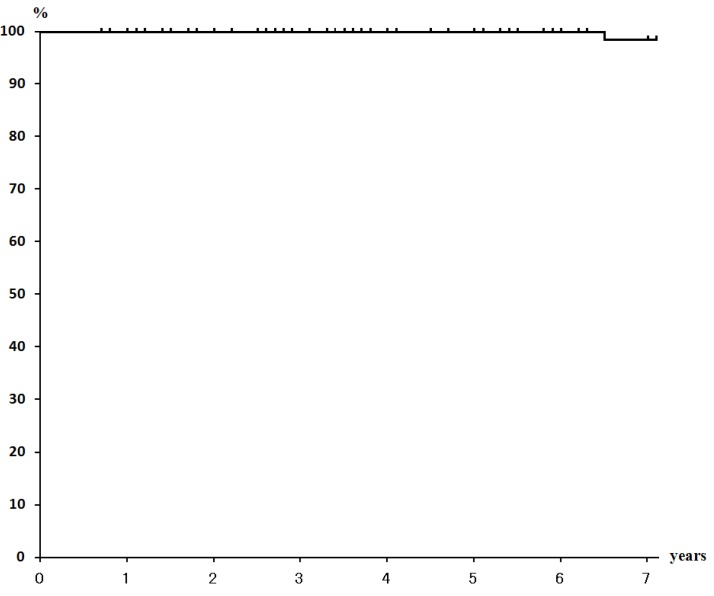
Overall Survival Rate (100% at 5 years).

**Figure 2:**
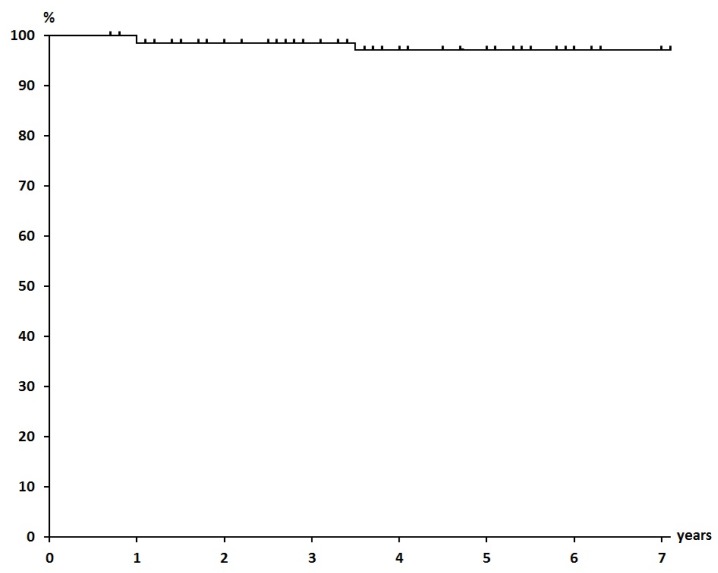
Survival Rate (NED) (97.1% at 5 years).

**Figure 3:**
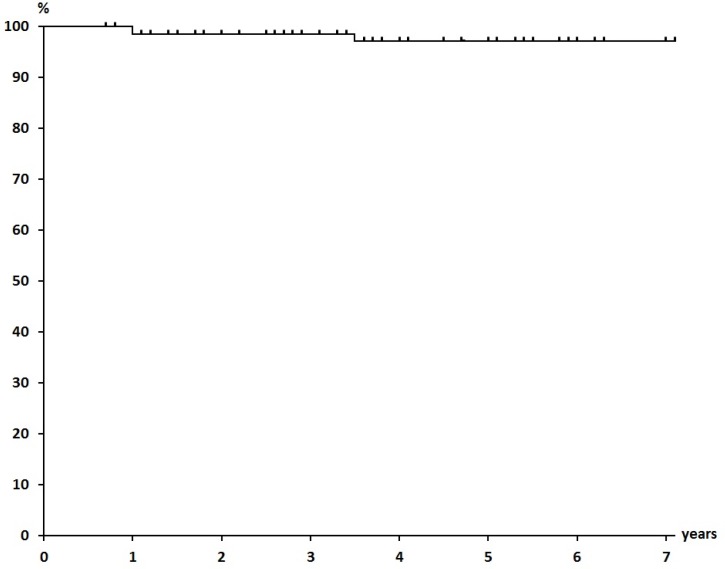
Local Control Rate (97.1% at 5 years).

The mean duration of follow-up as of the end of September 2014 was 51.1 months, at which time 68 of 70 patients were alive with no evidence of disease. Only one patient who had bilateral breast cancers, had local recurrence, which was discovered after 34 months of follow-up, and she died of cardiac failure at 86 years of age. Another patient had a solitary bone metastasis at Th12. The 5-year overall survival rate, NED rate, and local control rate were 100%, 97.1%, and 97.1%, respectively (Figure 1, Figure 2 and Figure 3).

**Figure 4:**
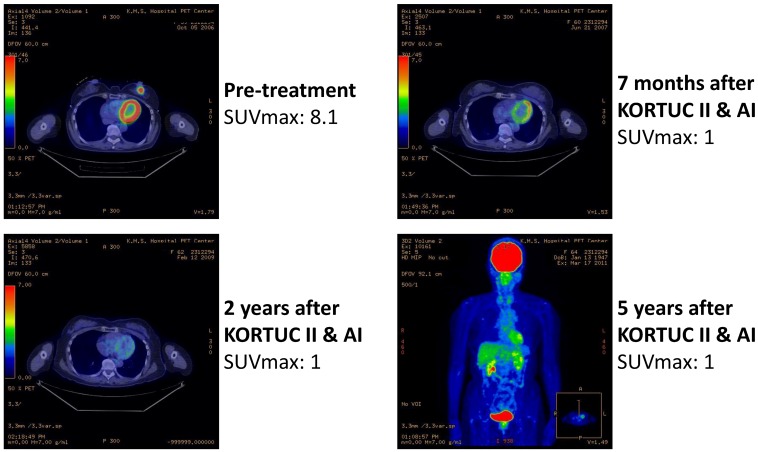
Case 4. 59 years old female patient with left breast cancer (cT2N0M0, ER: +, PgR: +, HER-2: -) treated with radiosensitization (KORTUC II) and without systemic chemotherapy. Left-upper: PET-CT image at pre-treatment. Tumor diameter is 27 mm and SUV-max value is 8.1; Right-upper: PET-CT image at 7 months following KORTUC II. Tumor is not recognized and SUV-max value on the region is approximately 1; Left-lower: PET-CT image at 2 years following KORTUC II. Tumor is not recognized and SUV-max value on the region is approximately 1; Right-lower: Whole body finding of PET-CT image at 5 years following KORTUC II. There is neither local recurrence nor distant metastasis.

**Figure 5:**
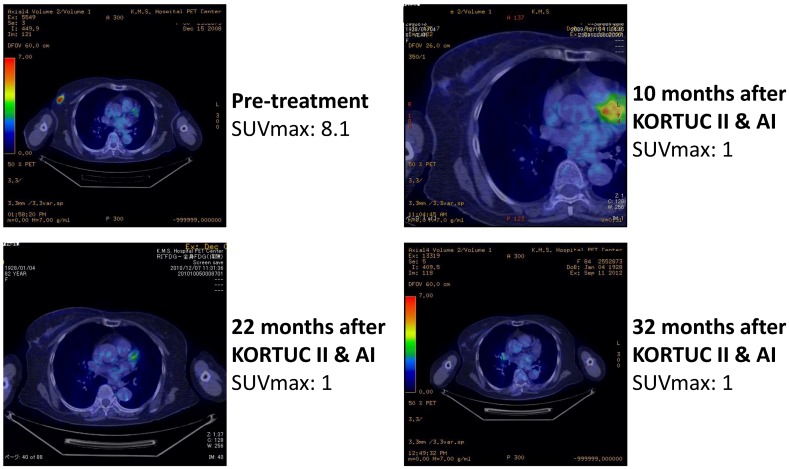
Case 18. 80 years old female patient with right breast cancer (cT2N1M0, ER: +, PgR: +, HER-2: ++ [FISH: -]) treated with radiosensitization (KORTUC II) and without systemic chemotherapy. Left-upper: PET-CT image at pre-treatment. Tumor diameter is 25 mm and SUV-max value is 8.1; Right-upper: PET-CT image at 10 months following KORTUC II. Tumor is not recognized and SUV-max value on the region is approximately 1; Left-lower: PET-CT image at 22 months following KORTUC II. Tumor is not recognized and SUV-max value on the region is approximately 1; Right-lower: PET-CT image at 32 months following KORTUC II. Tumor is not recognized and SUV-max value on the region is approximately 1.

**Figure 6:**
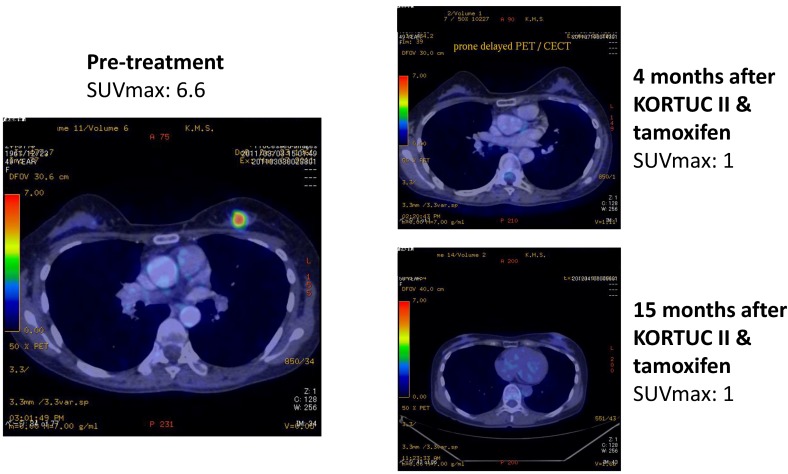
Case 40. 49 years old female patient with left breast cancer (cT2N0M0, ER: +, PgR: +, Ki-67 index: 24%) treated with radiosensitization (KORTUC II) and without systemic chemotherapy. Left: PET-CT image at pre-treatment. Tumor diameter is 24 mm and SUV-max value is 6.6; Right-upper: PET-CT image at 4 months following KORTUC II. Tumor is not recognized and SUV-max value on the region is approximately 1; Right-lower: PET-CT image at 15 months following KORTUC II. Tumor is not recognized and SUV-max value on the region is approximately 1.

**Figure 7:**
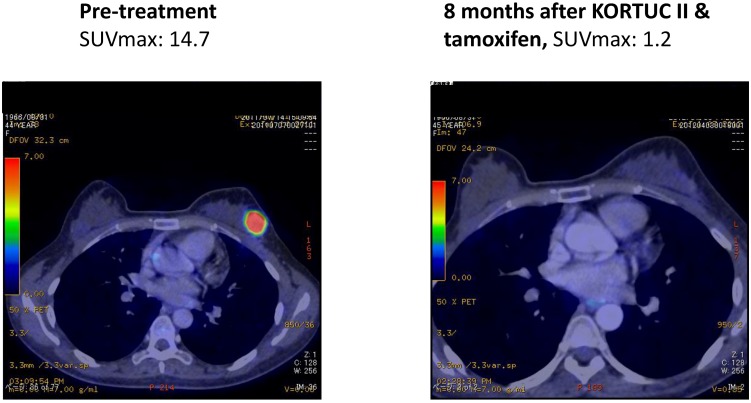
Case 44. 44 years old female patient with left breast cancer (cT2N0M0, ER: +, PgR: +, HER-2: 1+, Ki-67 index: 13%) treated with radiosensitization (KORTUC II) and without systemic chemotherapy. Left: PET-CT image at pre-treatment. Tumor diameter is 35 mm and SUV-max value is 14.7. Right: PET-CT image at 8 months following KORTUC II. Tumor is not recognized and SUV-max value on the region is approximately 1.2.

**Figure 8:**
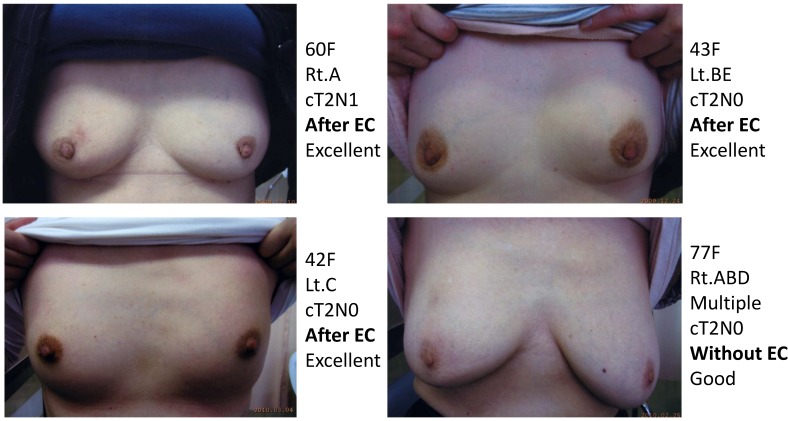
Photographs of examples of the outer appearances of the chest and breast of patients at approximately one year after KORTUC II treatment. Only one patient with multifocal breast cancer, shown in the right-lower image, had a cosmetic result that was evaluated as good. As for the three other patients, cosmetic results for the breast were evaluated as excellent.

As another acute toxicity related to KORTUC II, 10 patients complained of local pain at the injection site early during the trial, and the pain continued for several hours following injection of the agent. On the basis of their experience of local pain, a combined use of approximately 0.5 mL of 1% lidocaine with the KORTUC injection was started as of March 2008, and since then, none of the remaining 62 patients has complained of strong local pain. Changes in the PET-CT findings of representative patients are shown in Figure 4, Figure 5, Figure 6 and Figure 7. Photographs of examples of the outer appearances of the chest and breast of patients at approximately one year after KORTUC II treatment are shown in Figure 8.

## 3. Discussion

At approximately four years of mean follow-up, one patient had died of cardiac failure, and another patient had bone metastasis. Therefore, the 5-year overall survival rate was 100%, and the disease-free survival rate was calculated as 97.1%, showing relatively good therapeutic results. As shown in Table 1, Asterisks * indicate the patients described in our previous paper [9], and data with longer follow-up period are shown in the study in terms of further confirming the therapeutic results of the treatment.

The evidence in Western countries that the extent of surgical resection does not affect survival rates of patients is now generally accepted. Especially for early-stage breast cancer, BCT composed of breast-conserving surgery and postoperative radiotherapy has recently become standard therapy also in Japan. Given this trend, non-surgical and minimally invasive treatments are essential for breast cancer therapy in the near future.

As non-surgical therapy for breast cancer, besides KORTUC radiosensitization treatment, focused ultrasonographic therapy (FUS) and radiofrequency ablation (RFA) are possible [10,11,12,13]. However, there are serious difficulties with these two methods. For example, FUS therapy is limited to small breast cancer with a mean diameter of less than 10 mm, and the fat tissue surrounding the breast cancer should be relatively thick. Therefore, patients for whom FUS therapy is indicated are rare. Given that for RFA there is both limitation of the size of the breast cancer and difficulty in controlling local pain caused by insertion of a thick needle for RFA on local anesthesia, patients are not expected to have much benefit from these procedures.

In these situations, though carbon ion therapy, whose therapeutic outcome is not affected by tumor hypoxia, is somewhat promising, there are enormous costs associated with constructing carbon beam facilities and inexperience with a use of the Bragg’s peak in breast cancer therapy, which is one of major characteristics of the beam.

For the reasons mentioned above, development of radiosensitizers that can augment the therapeutic efficacy of X-rays and electrons has been awaited.

KORTUC II is a totally new modality of enzyme-targeting radiosensitization treatment developed at Kochi University, Japan, and the first agent for radiosensitization for intratumoral injection [1,2]. In KORTUC II treatment, conventionally used linear accelerators (Linacs), of which there are more than 1000 machines in Japan, can be used. In KORTUC, a mixed solution of hydrogen peroxide and sodium hyaluronate inactivates the anti-oxidative enzyme peroxidase in tumor tissue. In this reaction, oxygen is produced by degradation of hydrogen peroxide, partial oxygen pressure is increased, and radicals that are produced in tumor cells by X-ray irradiation are oxidized. Moreover, with the shortage of peroxidase activity in tumor cells, hydrogen peroxide, which is the last product of radicals and reactive-oxygen species, cannot be degraded and accumulates in the cell, and mitochondrial and lysosomal apoptosis of tumor cells is easily induced [14,15,16,17].

By the mechanism mentioned above, KORTUC can convert low-LET (linear energy transfer) radioresistant cells into radiosensitive ones [18,19], and it is considered to be an essential method to strengthen the therapeutic effect of radiotherapy using Linac.

KORTUC is a very safe method of radiosensitization in terms of application of the essential body components of hydrogen peroxide and sodium hyaluronate to augment the therapeutic effect of radiotherapy and/or chemotherapy. Actually, hydrogen peroxide exists in saliva, and sodium hyaluronate exists in subdermal tissue, *etc.* In fact, it has been approximately 50 years since the arterial injection of 250 mL of 0.12% hydrogen peroxide for radiotherapy of patients with head and neck cancer in 1967 [20]. Furthermore, KORTUC is an enzyme-targeting therapy, since the target is the anti-oxidative enzymes, peroxidase and catalase, as the main anti-oxidative enzymes in the human body. Therefore, KORTUC targets an essential defense system of tumor cells.

Moreover, in terms of intratumoral injection of the agent under ultrasonographic or CT guidance, the Bragg^,^s peak of the heavy particle beam, such as a carbon beam, is considered to be captured by imaging-guided injection of the radiosensitizer. It is one of the major characteristics of KORTUC that sodium hyaluronate, which was mainly developed in Japan, is used in the agent to increase viscosity in terms of preserving partial oxygen pressure in the tumor tissue.

In this procedure, the characteristics of radiation clinics in Japan, in which radiation oncologists and diagnostic radiologists usually belong to the same radiology department, are fully utilized. It is interesting to note that an image-guided radiosensitizing method such as KORTUC II, in which radiation oncologists and diagnostic radiologists work co-operatively, cannot be invented in Western countries, in which radiation oncologists belong to the cancer therapy division, and diagnostic radiologists belong to the diagnostic division, and they work completely separately. KORTUC can increase the direct social contribution of both radiation oncologists and diagnostic radiologists. Namely, as modern radiation oncologists take much time to perform complicated and precise radiation treatment planning (RTP), the time period for consulting with patients is greatly decreased. Under such circumstances, it might be a great aid for radiation oncologists to build good relationships with their patients by having them inject the radiosensitizer into their patients’ tumors twice a week, in co-operation with diagnostic radiologists.

Moreover, unlike research on radiosensitivity-related genes, which consumes enormous cost, KORTUC is considered to be a very favorable procedure in that it incurs less costs, maximally utilizing radical reactions, which compose two-thirds of the therapeutic effect of Linac.

With respect to radiosensitization using hydrogen peroxide, as mentioned above, there have been no reports for approximately these last 50 years following the report of intra-arterial injection of hydrogen peroxide published approximately 50 years ago [20]. The reason for this may be that the agent could not be precisely injected into the tumor tissue because there was no ultrasonographic equipment or CT then. Moreover, intra-arterial injection of an excess amount of hydrogen peroxide can result in oxygen embolism from oxygen produced by degradation of hydrogen peroxide by peroxidase in red blood cells and white blood cells. For the reason mentioned above, also in KORTUC II, it is considered risky and contraindicated to inject an excess amount of the agent into the great vessels. Therefore, injection of the agent intra-tumorally should be performed under imaging guidance. Intra-tumoral injection of KORTUC II is enabled by the development of scientific technology, mainly including modern diagnostic imaging such as power Doppler ultrasonography and MDCT (multi-row detector CT). Concerning KORTUC II, it has been patented in many leading countries, including Japan, UK, Germany, France, Australia, Canada and China.

The most important point of KORTUC II is to inject the agent intra-tumorally with a homogeneous distribution, using a relatively fine needle such as a 23-G needle, under imaging guidance, mainly ultrasonographic. The key characteristic of this treatment is that every radioresistant tumor is converted into a radiosensitive one in the tumor area where the agent is homogeneously injected.

When using the agent, it is essential to avoid direct injection of the agent into blood vessels and to confirm even distribution of oxygen microbubbles throughout the tumor tissue using ultrasonographic or CT guidance. Hence, KORTUC II can be considered a new imaging-guided, enzyme-targeted radiosensitization method. Because KORTUC II is safe and effective, as well as remarkably less expensive than other methods, it has great potential to become a viable non-invasive replacement for surgical procedures and a valuable radiosensitization method for most of low LET-radioresistant neoplasms.

## 4. Patients and Methods

A new radiosensitizing agent containing 0.5% hydrogen peroxide and 0.83% sodium hyaluronate (a CD44 ligand) has been developed for intra-tumoral injection into various tumors. This new method, named KORTUC II, was approved by our local ethics committee of Kochi Medical School for the treatment of breast cancer, metastatic lymph nodes, malignant melanoma, and soft tissue sarcoma, *etc*.

A total of 72 early-stage breast cancer patients (stage 0, 1 patient; stage I, 23; stage II, 48) were enrolled in the KORTUC II trial after providing fully informed consent at Kochi Medical School Hospital between October 2006 and September 2013 (Table 1). The patients’ mean age was 59.7 years, and they were all female. Patients were eligible for the study if they had an early-stage breast cancer and had either contraindications to general anesthesia due to significant comorbidity or had declined surgical treatment, and requested KORTUC II treatment.

A risk category was assigned to each patient according to the updated St.Gallen consensus based on clinical tumor size and the pathological results of a core needle biopsy taken before therapy.

After obtaining written, informed consent from the patients, non-surgical radiosensitization treatment with KORTUC II was performed, and the therapeutic effect was evaluated by PET-CT, breast MRI, ultrasonographic examination, and mammography at approximately 4 months and approximately 10 months after KORTUC II, and these examinations were repeated biannually for at least 5 years after KORTUC II treatment, thereafter.

For radiation therapy, treatment planning was performed using Pinnacle^3^, and hypofraction radiotherapy was administered using a tangential fields approach including an ipsilateral axillary region and field-in-field method. The energy level was 4 MV, and the total dose was 44 Gy administered as 2.75 Gy/fraction. An electron boost of 3 Gy was added three times just following the 14th, 15th, and 16th administrations of X-ray irradiation, using an electron beam of appropriate energy for each individual patient.

A maximum of 6 mL (usually 3 mL for tumors of less than approximately 3 cm in diameter) of the agent consisting of 0.5% hydrogen peroxide and 0.83% sodium hyaluronate was injected into breast tumor tissue twice a week (Monday and Thursday) under ultrasonographic guidance, just prior to each administration of radiation therapy. The injection was started immediately prior to the 6th fraction of radiation therapy to avoid possible increased migration of viable tumor cells into microvessels surrounding tumor tissue.

Concerning intratumoral injection of the agent, the injection was performed gradually, moving the depth and direction of the needle tip to obtain homogeneous distributions of microbubbles throughout the tumor under ultrasonographic guidance. At approximately three hours after injection of the agent, a CT study was occasionally performed to ascertain the distribution of microbubbles of oxygen produced by degradation of hydrogen peroxide by the inactivation of the anti-oxidative enzyme peroxidase.

From a needle biopsy specimen obtained at pre-treatment, hormonal status (estrogen and progesterone receptors), HER-2 antigen, the Ki-67 index, and CD44 receptor status were examined by immunohistochemistry. CD44-positive tumor cells have been reported to be breast cancer stem cells, and these cells might migrate into lymphatic vessels if sodium hyaluronate alone were injected into tumor tissue. Since hydrogen peroxide is utilized in KORTUC II treatment, the partial oxygen pressure in hypoxic breast cancer stem cells can be increased, and the radio-resistance of breast cancer stem cells under hypoxic circumstances is converted into a radio-sensitive state.

### 4.1. Formulation Example

A syringe (2.5 mL) of a hyaluronic acid preparation having a 1% *w*/*v* concentration of sodium hyaluronate (ARTZ Dispo, Seikagaku Corporation, Tokyo, Japan) was used. This contained 25 mg of sodium hyaluronate, 2.5 mg of L-methionine, sodium chloride, potassium phosphate, crystalline sodium dihydrogen phosphate, and an isotonizing agent. The preparation is a colorless, transparent, viscous, aqueous solution having a pH of 6.8 to 7.8, specific osmotic pressure of 1.0 to 1.2 (relative to physiological saline), and a weight-average molecular weight of 600,000 to 1.2 million. To this, 0.5 mL of a 3% *w*/*v* solution of hydrogen peroxide (Oxydol, Ken-ei Pharmaceutical Co. Ltd., Osaka, Japan) was added immediately before use and mixed well to prepare the radiosensitizer. Hydrogen peroxide, as a small vial containing 0.5 mL of 3% hydrogen peroxide, was aseptically produced and kindly provided to us by the Department of Pharmacy, Kochi Medical School Hospital. The sensitizer has a sodium hyaluronate concentration of 0.83% and a hydrogen peroxide concentration of approximately 0.5%. This preparation was used in the study.

### 4.2. Endocrine Therapy

All patients with hormone receptor-positive breast tumors received endocrine therapy immediately after the completion of KORTUC II treatment. Tamoxifen (20 mg/day per os) or an aromatase inhibitor (anastrozole 1 mg/day or exemestane 25 mg/day per os) was used for premenopausal and postmenopausal patients, respectively. Endocrine therapy was scheduled to continue for 5 years in all eligible patients.

### 4.3. Patient Assessment (Primary Breast Tumor and Toxicity of Therapy)

Tumor response was assessed according to the Revised Response Evaluation Criteria In Solid Tumors (RECIST) criteria [21] using CE-breast magnetic resonance imaging (MRI), FDG-positron emission tomography-computed tomography (PET-CT), and US. Patients were assigned a toxicity grade from a standard assessment scale (NIH common toxicity criteria). Treatment-related complications were assessed in detail in order to evaluate the feasibility of this approach. The patients underwent PET-CT and/or dynamic MRI examinations before and approximately 4 months after KORTUC II treatment, and every 6 months thereafter if possible for at least 5 years. Therapeutic effects were evaluated by comparing the results of pre and post PET-CT and MRI examinations of the treated parts. The final therapeutic response of the lesion was assessed according to the RECIST guidelines (version 1.1), and patient monitoring and tumor assessment were performed once a month. Patients were assigned a toxicity grade from a standard assessment scale (National Institutes of Health Common Toxicity Criteria). Treatment-related complications were assessed in detail to evaluate the feasibility of the approach according to the Common Terminology Criteria for Adverse Events (CTCAE criteria Version 4.0). Patients were followed for at least 12 months.

CE-breast MRI was performed at 3.0 T (Signa EXCITE HDx; GE Healthcare, Fairfield, CT, USA) with subjects in the prone position. Dynamic MRI using a three-dimensional fast spoiled gradient-echo sequence (VIBRANT, volume imaging for breast imaging; TR 7.0 ms; TE 4.0 ms; flip angle 10°; FOV 36 × 36 cm; matrix 512 × 256; slice thickness 3 mm; increment 0 mm; NEX 0.7) was obtained before and 8 times after (every 30 s) a bolus injection of 0.1 mmol/kg gadolinium-diethylenetriamine pentaacetic acid at a rate of 3 mL/s. Whole-body FDG-PET-CT scans were obtained on a Discovery ST Elite PET-CT system (GE Healthcare) consisting of a full ring dedicated PET and a 16-slice spiral CT. All patients were instructed to fast for 6 h before receiving an intravenous application of 3.5 MBq/kg FDG. Imaging was initiated ~60 min after injection of FDG. CT was acquired before PET with 50 mA/s at 130 kV without administration of a non-ionic contrast agent. All images were reconstructed with a 5-mm slice thickness and a 3.7-mm increment. After CT, a 3-D mode PET was performed. The PET emission time per bed position was adapted to the patient body weight: <65 kg, 2 min per bed position; 65–85 kg, 2.5 min; and >85 kg, 3 min. Any focally elevated PET signal above normal that could be mapped to a tumor location was rated as positive for viable breast cancer. The interpreters of ultrasonographic (K.K.), CE-breast MRI (K.K., N.H.), and FDG-PET-CT (M.N.) examinations were provided information regarding tumor location, but they were otherwise blinded to patient and therapy information.

### 4.4. Beginning and Frequency of Observation

Assessment of the primary tumor started within 16 weeks of the completion of KORTUC II treatment, regardless of the endocrine therapy. CE-breast MRI and FDG-PET-CT were performed at least once a year following the completion of KORTUC II treatment. Ultrasonographic and clinical examinations were performed every 3 months.

## 5. Conclusions

Non-surgical BCT can be performed using KORTUC II, which has three major characteristics: imaging guidance by ultrasonography; enzyme-targeting of peroxidase/catalase; and targeting of breast cancer stem cells via the CD44 receptor.

Local control and cosmesis have remained excellent at the most recent follow-up, with acceptable rates of acute/late toxicities. Non-surgical BCT (KORTUC-BCT) can be performed safely and effectively for patients with stage I or II breast cancer. To demonstrate this clearly, it is essential to conduct randomized clinical trials in Western countries, as well as in Japan. Our goal is to establish and widely promote KORTUC-BCT, non-surgical chemo-radiosensitization treatment for locally advanced breast cancer (KORTUC-LABC), electron-radiosensitization for lesions with local recurrence (KORTUC-REC), including post-radiotherapy lesions, transcatheter arterial chemo-sensitizing embolization treatment (KORTUC-TACE), and intra-operative radiosensitization treatment for locally-advanced stage IVa pancreatic carcinoma (KORTUC-IOR).

## References

[1] Ogawa Y., Kubota K., Ue H., Kataoka Y., Tadokoro M., Miyatake K., Tsuzuki K., Yamanishi T., Itoh S., Hitomi J. (2009). Phase I study of a new radiosensitizer containing hydrogen peroxide and sodium hyaluronate for topical tumor injection: A new enzyme-targeting radiosensitization treatment, Kochi Oxydol-Radiation Therapy for Unresectable Carcinomas, Type II (KORTUC II). Int. J. Oncol..

[2] Ogawa Y., Kubota K., Ue H., Tadokoro M., Matsui R., Yamanishi T., Hamada N., Kariya S., Nishioka A., Nakajima H. (2011). Safety and effectiveness of a new enzyme-targeting radiosensitization treatment (KORTUC II) for intratumoral injection for low-LET radioresistant tumors. Int. J. Oncol..

[3] Clarke M., Collins R., Darby S., Davies C., Elphinstone P., Evans V., Godwin J., Gray R., Hicks C., James S. (2005). Effects of radiotherapy and of differences in the extent of surgery for early breast cancer on local recurrence and 15-year survival: An overview of the randomized trials. Lancet.

[4] Punglia R., Morrow M., Winer E.P., Harris J.R. (2007). Local therapy and survival in breast cancer. N. Engl. J. Med..

[5] Cabanes P.A., Salmon R.J., Vilcoq J.R., Durand J.C., Fourquet A., Gautier C., Asselain B. (1992). Value of axillary dissection in addition to lumpectomy and radiotherapy in early breast cancer. The Breast Carcinoma Collaborative Group of the Institut Curie. Lancet.

[6] Green M.C., Buzdar A.U., Smith T., Ibrahim N.K., Valero V., Rosales M.F., Cristofanilli M., Booser D.J., Pusztai L., Rivera E. (2005). Weekly paclitaxel improves pathologic complete remission in operable breast cancer when compared with paclitaxel once every 3 weeks. J. Clin. Oncol..

[7] Mukai H., Watanabe T., Mitsumori M., Tsuda H., Nakamura S., Masuda N., Yamamoto N., Shibata T., Sato A., Iwata H. (2013). Final results of a safety and efficacy trial of preoperative sequential chemoradiation therapy for the nonsurgical treatment of early breast cancer: Japan Clinical Oncology Group Study JCOG0306. Oncology.

[8] Hall E.J. (2000). The oxygen effect and reoxygenation. Radiobiology for the Radiologist.

[9] Tsuzuki A., Ogawa Y., Kubota K., Tokuhiro S., Akima R., Yaogawa S., Itoh K., Yamada Y., Sasaki T., Onogawa M. (2011). Evaluation of changes in tumor shadows and microcalcifications on mammography following KORTUC II, a new radiosensitization treatment without any surgical procedure for elderly patients with stage I and II breast cancer. Cancers.

[10] Schmitz A.C., Gianfelice D., Daniel B.L., Mali W.P., van den Bosch M.A. (2008). Image-guided focused ultrasound ablation of breast cancer: Current status, challenges, and future directions. Eur. Radiol..

[11] Jolesz F.A. (2009). MRI-guided focused ultrasound surgery. Annu. Rev. Med..

[12] Manenti G., Bolacchi F., Perretta T., Cossu E., Pistolese C.A., Buonomo O.C., Bonanno E., Oriandi A., Simonetti G. (2009). Small breast cancers: *In vivo* percutaneous US-guided radiofrequency ablation with dedicated cool-tip radiofrequency system. Radiology.

[13] Kinoshita T., Iwamoto E., Tsuda H., Seki K. (2011). Radiofrequency ablation as local therapy for early breast carcinomas. Breast Cancer.

[14] Ogawa Y., Takahashi T., Kobayashi T., Kariya S., Nishioka A., Mizobuchi H., Noguchi M., Hamasato S., Tani T., Seguchi H. (2003). Mechanism of apoptotic resistance of human osteosarcoma cell line, HS-Os-1, against irradiation. Int. J. Mol. Med..

[15] Tokuhiro S., Ogawa Y., Tsuzuki K., Akima R., Ue H., Kariya S., Nishioka A. (2010). Development of a new enzyme-targeting radiosensitizer (KORTUC) containing hydrogen peroxide for intratumoral injection for patients with low linear energy transfer (LET) radioresistant neoplasms. Oncol. Lett..

[16] Ogawa Y., Takahashi T., Kobayashi T., Kariya S., Nishioka A., Hamasato S., Moriki T., Seguchi H., Yoshida S., Sonobe H. (2004). Immunocytochemical characteristics of human osteosarcoma cell line HS-Os-1: Possible implication in apoptotic resistance against irradiation. Int. J. Mol. Med..

[17] Ogawa Y., Takahashi T., Kobayashi T., Toda M., Nishioka A., Kariya S., Seguchi H., Yamamoto H., Yoshida S. (2003). Comparison of radiation-induced reactive oxygen species formation in adult articular chondrocytes and that in human peripheral T cells: Possible implication in radiosensitivity. Int. J. Mol. Med..

[18] Ogawa Y., Takahashi T., Kobayashi T., Kariya S., Nishioka A., Ohnishi T., Saibara T., Hamasato S., Tani T., Seguchi H. (2003). Apoptotic-resistance of the human osteosarcoma cell line HS-Os-1 to irradiation is converted to apoptotic-susceptibility by hydrogen peroxide: A potent role of hydrogen peroxide as a new radiosensitizer. Int. J. Mol. Med..

[19] Kariya S., Sawada K., Kobayashi T., Karashima T., Shuin T., Nishioka A., Ogawa Y. (2009). Combination treatment of hydrogen peroxide and X-rays induces apoptosis in human prostate cancer PC-3 cells. Int. J. Radiat. Oncol. Biol. Phys..

[20] Chasin W.D., Gross C.C., Wang C.C., Miller D. (1967). Hydrogen peroxide and irradiation of tumors. Arch. Otolaryngol..

[21] Therasse P., Arbuck S.G., Eisenhauer E.A., Wanders J., Kaplan R.S., Rubinstein L., Verweij J., van Glabbeke M., van Oosterom A.T., Christian M.C. (2000). New guidelines to evaluate the response to treatment in solid tumors. European Organization for Research and Treatment of Cancer, National Cancer Institute of the United States, National Cancer Institute of Canada. J. Natl. Cancer Inst..

